# Supplementary material for: Causal Relationship between Aging and Anorexia Nervosa: A White-Matter-Microstructure-Mediated Mendelian Randomization Analysis
Source: Biomedicines. 2024 Aug 16;12(8):1874. doi: 10.3390/biomedicines12081874 (PMC11351342; doi:10.3390/biomedicines12081874)
Supplement: Supplementary file 1 [file biomedicines-12-01874-s001.zip › Supplementary Figure/Figure S8/Leaveoneout plot of MR analysis for TL to ubm-b-1606.pdf]

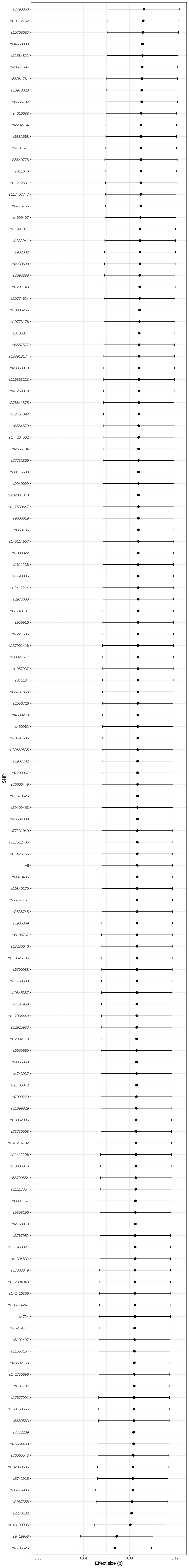
rs7790856
rs10112752
rs10768683
rs34550383
rs11584821
rs28577594
rs56061761
rs16978028
rs6536702
rs4616688
rs2282764
rs6881568
rs4731541
rs35640778
rs611646
rs11212631
rs117407747
rs6776756
rs4695407
rs11991877
rs1332941
rs932002
rs2230590
rs3093888
rs1291143
rs10774624
rs10905255
rs10773176
rs3785074
rs6587577
rs188918174
rs28363070
rs116863223
rs41269079
rs376641875
rs12451892
rs6584579
rs144204502
rs2555104
rs77732866
rs80116508
rs9940099
rs202034370
rs117630647
rs9600019
rs869785
rs145114957
rs1003322
rs1611236
rs4498805
rs12412214
rs2977608
rs61748181
rs939916
rs7221585
rs137901416
rs80324517
rs1957937
rs871134
rs66731853
rs2056726
rs4530278
rs450962
rs78491606
rs139669835
rs1907702
rs7209057
rs76666449
rs11579626
rs59409453
rs45604339
rs77231040
rs117512405
rs11426156
All
rs9878436
rs10840270
rs55747751
rs2538745
rs1985369
rs8105767
rs13230646
rs113525195
rs6790988
rs11769630
rs10845387
rs7164950
rs117034449
rs12925933
rs12932179
rs6659669
rs9955360
rs4743037
rs61405042
rs7099229
rs11699829
rs13062095
rs73730598
rs141214782
rs11412296
rs10805346
rs56799554
rs11117354
rs3891167
rs9398196
rs2763979
rs3767952
rs111950327
rs41304832
rs17803849
rs112394943
rs142426306
rs185174247
rs4724
rs76219171
rs8102497
rs11557154
rs28502153
rs142730696
rs131797
rs17677991
rs150150565
rs6669563
rs7772289
rs75664430
rs76065543
rs182059586
rs5742915
rs35446936
rs2967355
rs2276182
rs143190905
rs9419958
rs7705526
SNP
0.00
0.04
0.08
0.12
Effect size (b)
